# Supplementary material for: The subthalamic nucleus contributes causally to perceptual decision-making in monkeys
Source: bioRxiv. 2024 Apr 13:2024.04.09.588715. Preprint. [Version 1] doi: 10.1101/2024.04.09.588715 (PMC11030388; doi:10.1101/2024.04.09.588715)
Supplement: Supplementary file 1 [file NIHPP2024.04.09.588715v1-supplement-1.pdf]

bioRxiv preprint doi: https://doi.org/10.1101/2024.04.09.588715; this version posted April 13, 2024. The copyright holder for this preprint (which was not certified by peer review) is the author/funder, who has granted bioRxiv a license to display the preprint in perpetuity. It is made available under aCC-BY 4.0 International license.

Supplemental Information

Suppl. Figure 1. STN activity is modulated by choice and RT. Same format as Figure 2, except using choice and RT as regressors.

Suppl. Figure 2. Clustering parameters. A, Silhouette plots for clustering results using different combinations of settings. Silhouette scores for neurons are grouped by clusters and sorted. Red lines indicate the mean scores. Yellow shaded box indicates the chosen setting for results in Figure 3. B, Average Rand indices for different clustering settings. For each setting, the k-means algorithm was run 50 times, each time picking the best clusters out of 100 repetitions. Higher Rand index indicates greater cluster stability across different runs. C, Mean silhouette scores and the number of negative scores as a function of number of clusters, using the firing rate vectors and correlation distance. Higher mean score and fewer negative scores indicate better clustering.

Suppl. Figure 3. Clustering results using alternative numbers of clusters, visualized in tSNE space. Same format as Figure 3E.

Suppl Figure 4. Comparison of different logistic models. A, The No Lapse model was associated with the lowest AIC for most sessions. The Symmetric Lapse model was associated with lower AICs for 12 sessions. The Asymmetric Lapse model was associated with lower AICs for 8 sessions. B, Histograms of microstimulation effects on bias, slope, and lapse terms in the Symmetric Lapse model. C, Histograms of microstimulation effects on bias, slope, and two lapse (for each choice) terms in the Asymmetric Lapse model. Same format as the histograms in Figure 6A.

Suppl Figure 5. A, Differences in AIC between reduced and Full models. Filled circles indicate sessions for which $AIC_{Reduced} - AIC_{Full} > 3$ (red line). Note that for three sessions, the Full model outperformed the None model but not any of the reduced models. B, Histograms of difference in DDM parameters between trials with and without microstimulation. Filled bars represent sessions considered to show significant microstimulation effects on the given parameter, based on AIC comparisons. Triangles indicate median values. Filled triangles: Wilcoxon sign-rank test, $p < 0.05$.

bioRxiv preprint doi: https://doi.org/10.1101/2024.04.09.588715; this version posted April 13, 2024. The copyright holder for this preprint (which was not certified by peer review) is the author/funder, who has granted bioRxiv a license to display the preprint in perpetuity. It is made available under aCC-BY 4.0 International license.

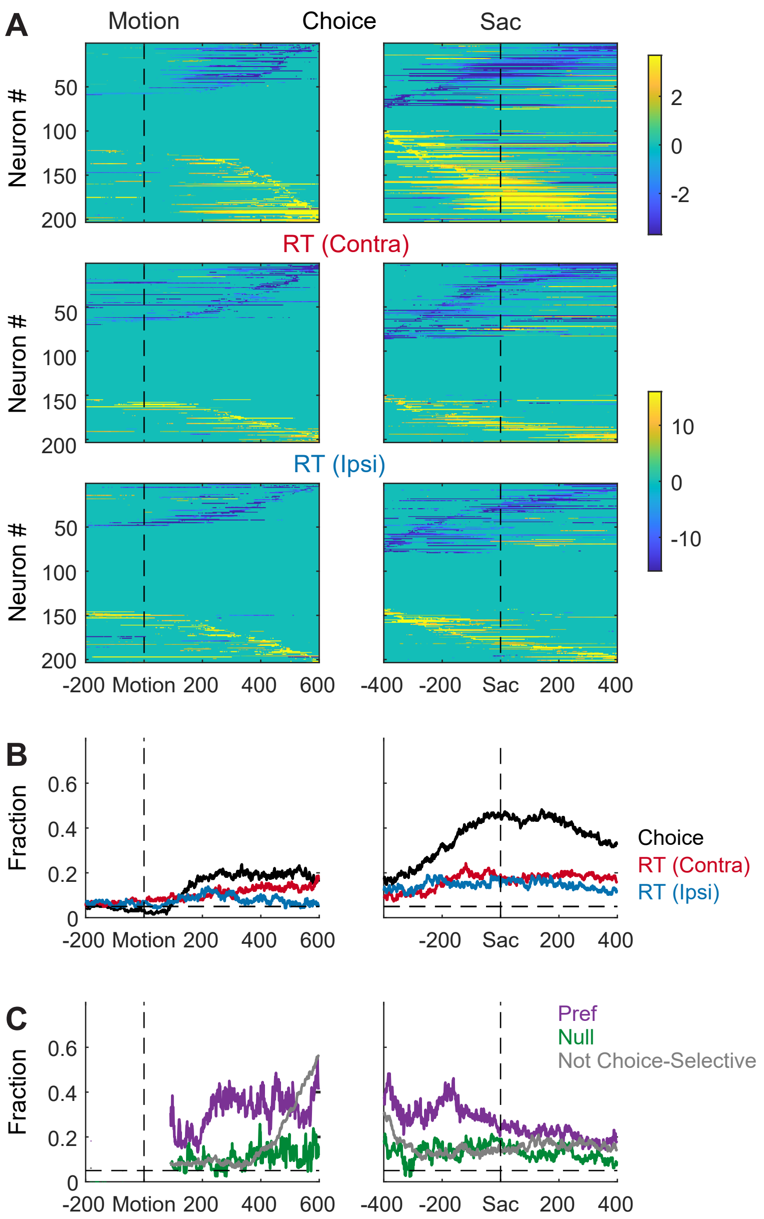

Suppl. Figure 1. STN activity is modulated by choice and RT. Same format as Figure 2, except using choice and RT as regressors.

bioRxiv preprint doi: https://doi.org/10.1101/2024.04.09.588715; this version posted April 13, 2024. The copyright holder for this preprint (which was not certified by peer review) is the author/funder, who has granted bioRxiv a license to display the preprint in perpetuity. It is made available under aCC-BY 4.0 International license.

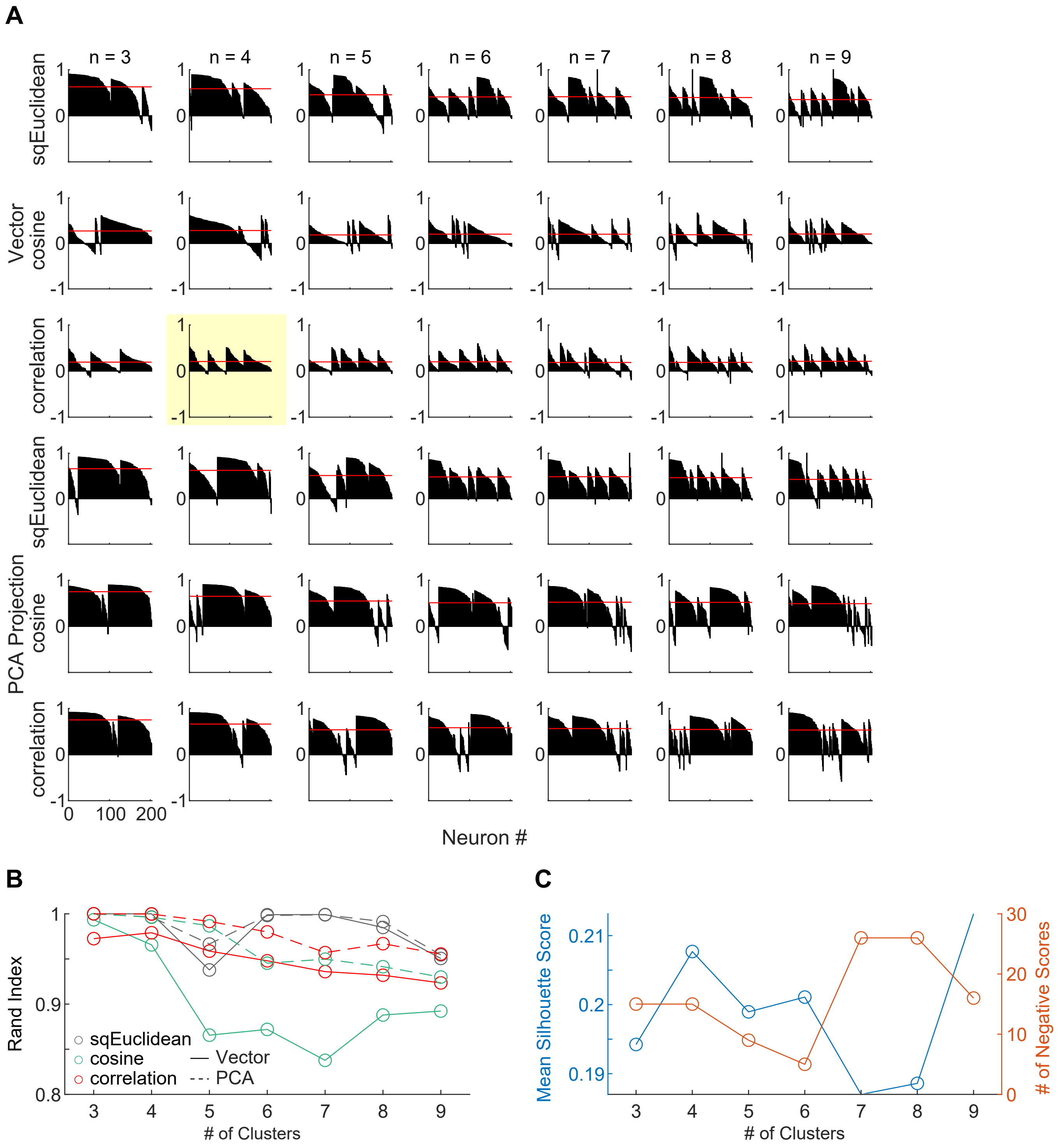

Suppl. Figure 2. Clustering parameters. A, Silhouette plots for clustering results using different combinations of settings. Silhouette scores for neurons are grouped by clusters and sorted. Red lines indicate the mean scores. Yellow shaded box indicates the chosen setting for results in Figure 3. B, Average Rand indices for different clustering settings. For each setting, the k-means algorithm was run 50 times, each time picking the best clusters out of 100 repetitions. Higher Rand index indicates greater cluster stability across different runs. C, Mean silhouette scores and the number of negative scores as a function of number of clusters, using the firing rate vectors and correlation distance. Higher mean score and fewer negative scores indicate better clustering.

bioRxiv preprint doi: https://doi.org/10.1101/2024.04.09.588715; this version posted April 13, 2024. The copyright holder for this preprint (which was not certified by peer review) is the author/funder, who has granted bioRxiv a license to display the preprint in perpetuity. It is made available under aCC-BY 4.0 International license.

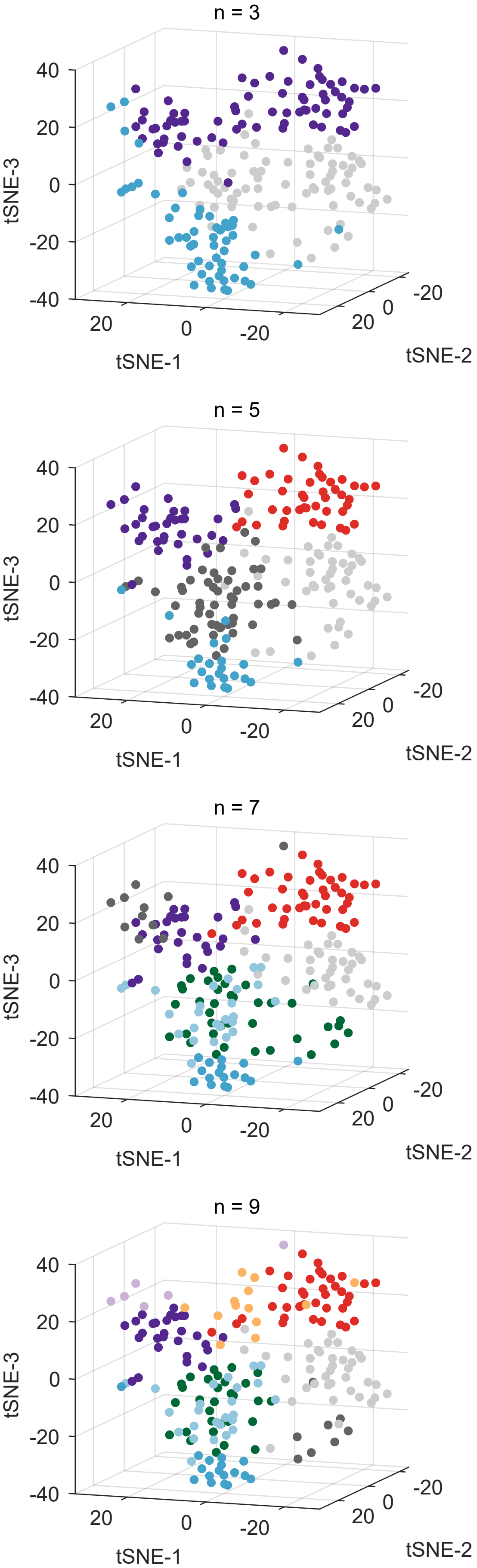

Suppl. Figure 3. Clustering results using alternative numbers of clusters, visualized in tSNE space. Same format as Figure 3E.

bioRxiv preprint doi: https://doi.org/10.1101/2024.04.09.588715; this version posted April 13, 2024. The copyright holder for this preprint (which was not certified by peer review) is the author/funder, who has granted bioRxiv a license to display the preprint in perpetuity. It is made available under aCC-BY 4.0 International license.

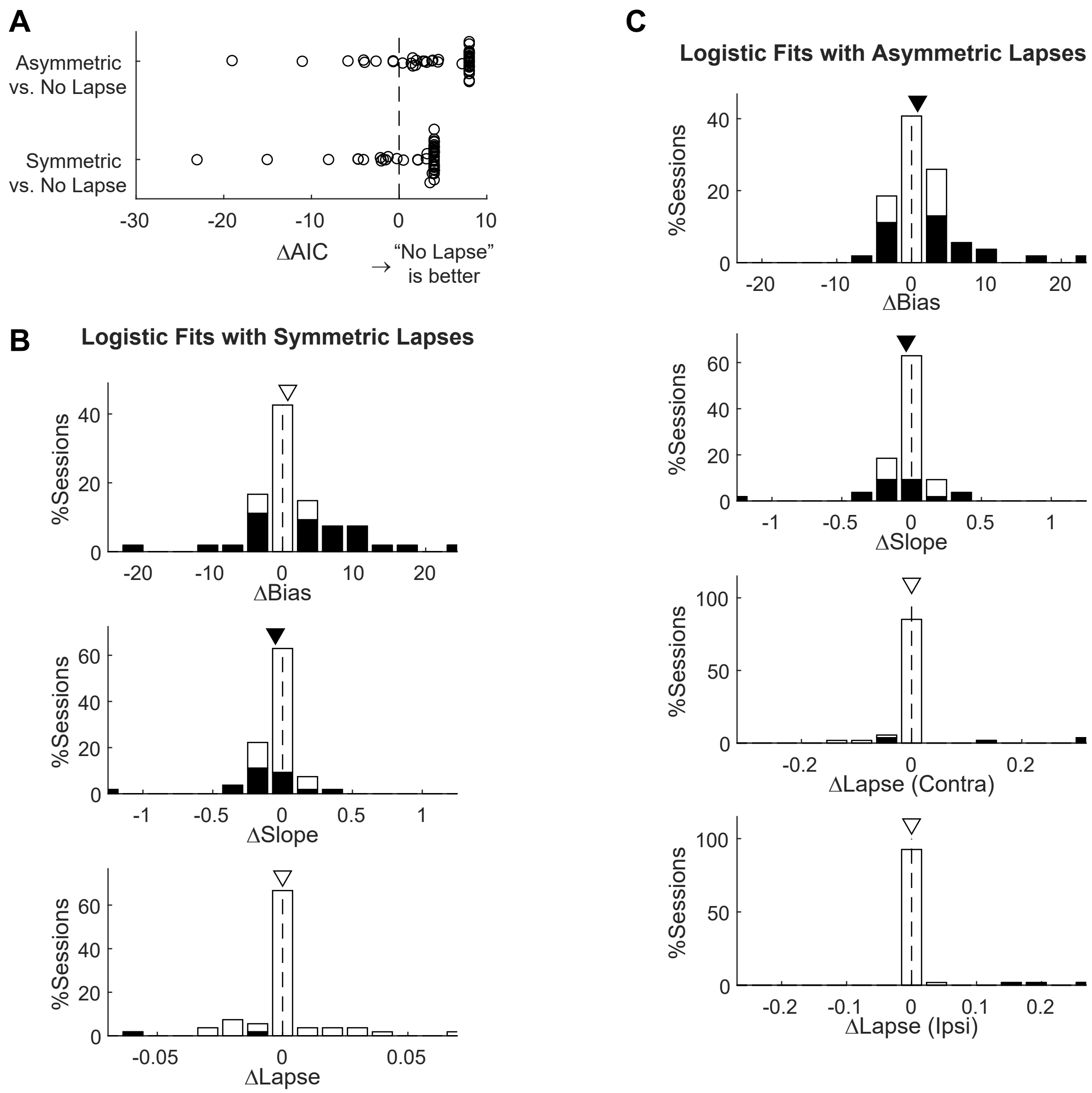

Suppl Figure 4. Comparison of different logistic models. A, The No Lapse model was associated with the lowest AIC for most sessions. The Symmetric Lapse model was associated with lower AICs for 12 sessions. The Asymmetric Lapse model was associated with lower AICs for 8 sessions. B, Histograms of microstimulation effects on bias, slope, and lapse terms in the Symmetric Lapse model. C, Histograms of microstimulation effects on bias, slope, and two lapse (for each choice) terms in the Asymmetric Lapse model. Same format as the histograms in Figure 6A.

bioRxiv preprint doi: https://doi.org/10.1101/2024.04.09.588715; this version posted April 13, 2024. The copyright holder for this preprint (which was not certified by peer review) is the author/funder, who has granted bioRxiv a license to display the preprint in perpetuity. It is made available under aCC-BY 4.0 International license.

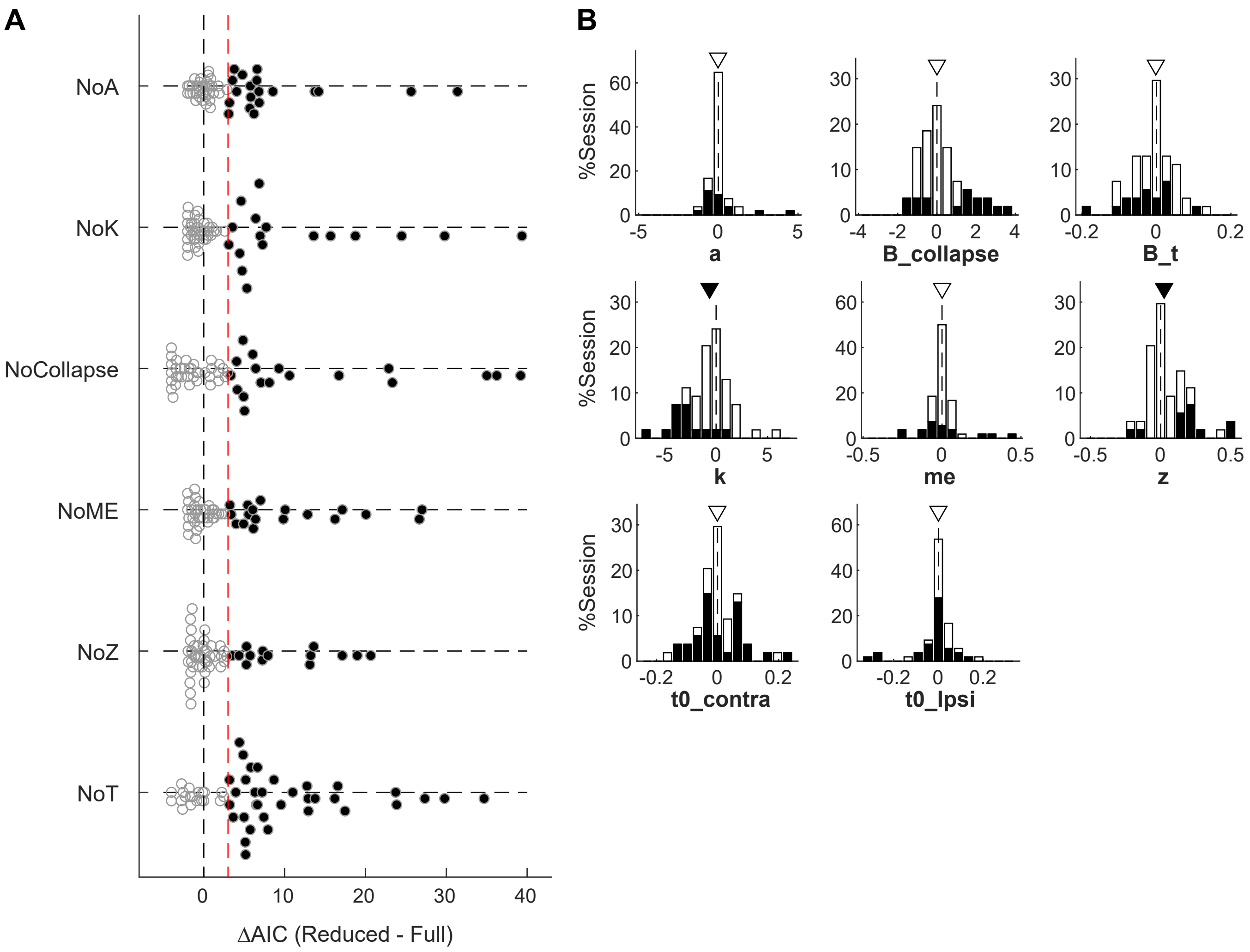

Suppl Figure 5. A, Differences in AIC between reduced and Full models. Filled circles indicate sessions for which $AIC_{Reduced} - AIC_{Full} > 3$ (red line). Note that for three sessions, the Full model outperformed the None model but not any of the reduced models. B, Histograms of difference in DDM parameters between trials with and without microstimulation. Filled bars represent sessions considered to show significant microstimulation effects on the given parameter, based on AIC comparisons. Triangles indicate median values. Filled triangles: Wilcoxon sign-rank test, $p < 0.05$.
